# High-Frequency Ultrasound Assessment of Basal Cell Carcinoma: Correlations Between Histopathological Subtype, Vascularity, and Age/Sex Distribution

**DOI:** 10.3390/cancers18020274

**Published:** 2026-01-15

**Authors:** Klára Szalai, Klaudia Tóth, Judit Hársing, Miklós Gyöngy, Péter Holló

**Affiliations:** 1 Department of Dermatology, Venereology and Dermatooncology, Semmelweis University, 1085 Budapest, Hungary; 2 Faculty of Information Technology and Bionics, Pázmány Péter Catholic University, 1088 Budapest, Hungary

**Keywords:** ultrasound, BCC, HFUS, oncology

## Abstract

**Background:** High-frequency ultrasound (HFUS) has emerged as a valuable non-invasive imaging modality for the preoperative assessment of basal cell carcinoma (BCC). However, its ability to reliably differentiate between histopathological subtypes based on morphological and vascular characteristics requires further validation. **Methods:** Between January 2010 and December 2011, 320 patients with a total of 330 histologically confirmed BCC lesions were examined using HFUS (15–18 MHz linear transducer). Lesions were classified according to ultrasound contour (sharp vs. irregular) and vascularity (hypervascular vs. hypovascular) and correlated with histopathological subtype (solid vs. infiltrative). Postoperative ultrasound follow-up was performed in a subset of patients for recurrence detection. **Results:** Solid BCCs were predominantly characterised by sharp, well-defined margins, whereas infiltrative tumours more frequently exhibited irregular contours. This association was highly significant (χ^2^ = 24.7, df = 1, *p* < 0.001; OR = 71.9, 95% CI: 37.0–139.8). Vascularity patterns also differed significantly between subtypes: solid tumours were more likely to present with hypervascular features, while infiltrative tumours more frequently exhibited hypovascular patterns (χ^2^ = 23.8, df = 1, *p* < 0.001; OR = 3.24). No statistically significant associations were observed between ultrasound morphology and patient sex or age. Among patients who participated in postoperative HFUS follow-up, seven histologically confirmed recurrences were detected. **Conclusions:** HFUS provides reliable preoperative information on BCC morphology and vascularity, enabling accurate differentiation between solid and infiltrative subtypes. These findings support the role of HFUS as a valuable adjunct to dermatoscopy in treatment planning and postoperative surveillance of BCC.

## Simple Summary

Basal cell carcinoma is the most common type of skin cancer and usually grows slowly, but some forms can behave more aggressively. High-frequency ultrasound (HFUS) is a non-invasive imaging method that supports the preoperative evaluation of basal cell carcinoma. In this study, ultrasound contour and vascularity patterns showed a strong association with histological subtype, enabling reliable differentiation between solid and infiltrative tumours. Solid lesions were typically well-defined and hypervascular, whereas infiltrative tumours more often showed irregular margins and reduced vascularity. HFUS therefore represents a valuable adjunct to dermatoscopy for treatment planning and postoperative follow-up.

## 1. Introduction

**Figure 1:**
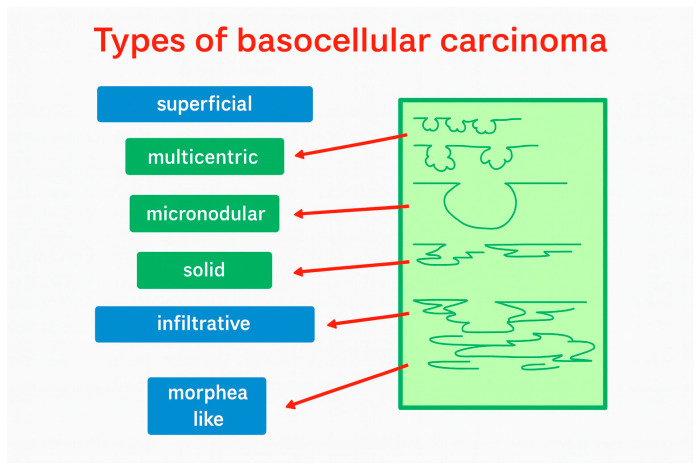
The schematic morphology of BCCs.

Basal cell carcinoma (BCC) is the most common type of malignant skin disease. While it rarely metastasizes, it can exhibit locally aggressive behavior, and recurrence is significantly influenced by treatment strategy. Since a large proportion of BCCs occur in the head and neck region, minimizing recurrence is critical. Optimizing surgical margins and tailoring follow-up based on histopathology and recurrence risk are essential [1]. Dermatoscopy is the gold standard for establishing the diagnosis of skin lesions; however, among other imaging modalities, ultrasound is indispensable for assessing the depth, vascularity, and morphology of these lesions [2]. Figure 1 shows the schematic morphology of BCCs.

**Figure 2:**
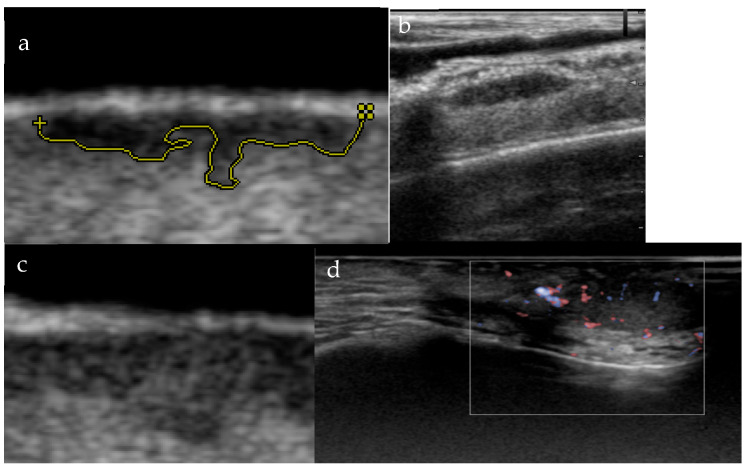
(**a**–**d**). US morphology of micronodular superficial infiltrative BCC (**a**). US morphology of solid nodular BCC (**b**). US morphology of infiltrative BCC (**c**). US morphology of recurrent infiltrative BCC (**d**).

High-frequency ultrasound (HFUS, 15–33 MHz) has become an increasingly valuable diagnostic tool [3,4]. According to the EFSUMB Position Paper, HFUS is defined as ≥15 MHz [5,6]. HFUS can delineate dermal structures, differentiate nodular and infiltrative subtypes, and evaluate vascularity and echogenicity [7]. Preoperative HFUS may help select treatment, reduce recurrence, and guide follow-up monitoring [6,8,9,10]. In the primary evaluation of the tumor, both the vertical and horizontal dimensions are critical for accurate determination of oncologic safety margins [11]. This assessment is contingent upon the frequency range of the ultrasound transducer, as higher frequencies allow more precise characterization of key parameters, particularly the depth of infiltration [12]. High-frequency probes, up to 15–20 MHz, provide optimal resolution for such diagnostic purposes [13]. Figure 2 shows the possible ultrasound morphology of BCC lesions in primary and recurrent cases.

**Figure 3:**
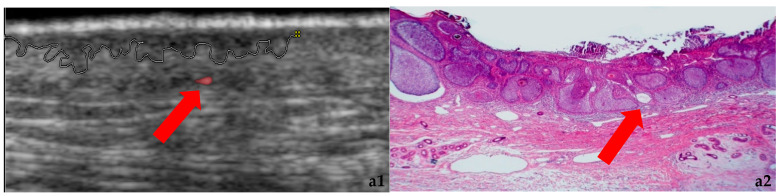

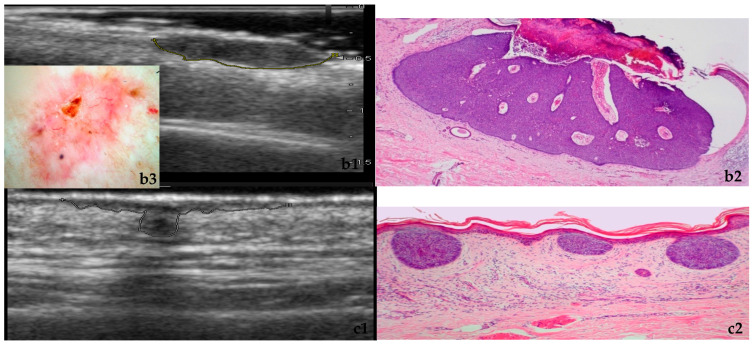
(**a1**–**c2**). Micronodular type: US morphology (**a1**) and histopathology (**a2**). Nodular type: US morphology (**b1**), histopathology (**b2**), and dermoscopy (**b3**). Micronodular superficial type: US morphology (**c1**) and histopathology (**c2**). Red arrow indicates the vessels.

Since the early 2010s, HFUS has complemented dermatoscopy in dermatology, allowing detailed assessment of BCC depth and morphology [14]. BCC subtypes typically show distinct ultrasound patterns: superficial, nodular, variants with nevoid or cystic components [14,15]. This study aims to describe ultrasound morphology of BCCs, correlate findings with histopathology, and evaluate sex- and age-related differences in lesion features to optimize preoperative planning and follow-up. Figure 3 illustrates the ultrasound morphology of different BCCs together with their dermatoscopic images.

## 2. Materials and Methods

### 2.1. Study Population and Design

Between 1 January 2010 and 31 December 2011, 320 patients with a total of 330 histologically confirmed basal cell carcinomas were examined using a Hitachi Preirus HR high-frequency ultrasound system (15–18 MHz linear transducer, REF EUP-L75). The study population included 157 female and 163 male patients (Ethical approval number: SE RKEB 16/2022). Examinations were performed in patients in whom basal cell carcinoma was newly detected by dermatoscopy. Lesions were located in the head and neck, extremity, and trunk regions; however, lesion localization was not analysed in the present study.

All examinations were performed using the same ultrasound system and transducer. All ultrasound examinations were performed by a single radiologist with extensive prior experience in dermatologic high-frequency ultrasound and routine correlation with histopathological findings before the start of the study period. The examiner had already completed several years of HFUS-based skin tumour imaging and histopathology-correlated diagnostics prior to January 2010, ensuring a stable level of diagnostic expertise throughout the study. The imaging protocol included B-mode imaging, color Doppler mode, and strain elastography. However, due to methodological limitations, strain elastography did not yield data specific to basal cell carcinoma within this patient cohort. Lesions were considered hypovascular when no signal was detected within the color Doppler box and hypervascular when at least three color signals were observed. Doppler waveforms were not generated. Using standard ultrasound terminology, lesions were characterised by sharp or irregular contours, whereas histopathological findings were classified into solid and infiltrative subtypes.

Lesions were categorized based on morphology (sharp vs. irregular contours) and vascularity (hypervascular vs. hypovascular) [16,17,18]. We assessed the patients’ sex and age, as well as the ultrasound morphology of the lesions, including their contours and vascularity, and compared these findings with the histopathological results and the intact anatomical margins.

During the ultrasound examination, measurements of the lesion’s maximal transverse diameter and depth extension were recorded in longitudinal and transverse planes. Tumour depth was defined as the maximum vertical distance from the epidermal surface to the deepest detectable tumour margin and ranged between 0.6 and 2.1 mm in the examined cohort. These depth parameters were documented systematically and used to support surgical margin planning. However, depth-related analyses are being evaluated separately in an ongoing study focusing on ultrasound–histopathology depth correlation and were therefore not included in the present analysis.

Postoperative HFUS assessments were performed at 3, 6, and 12 months and then annually, extending until 2024. Recurrences were recorded. Follow-up intervals were adjusted based on patient symptoms, dermatoscopic findings, and histopathological results [17,18].

**Figure 4:**
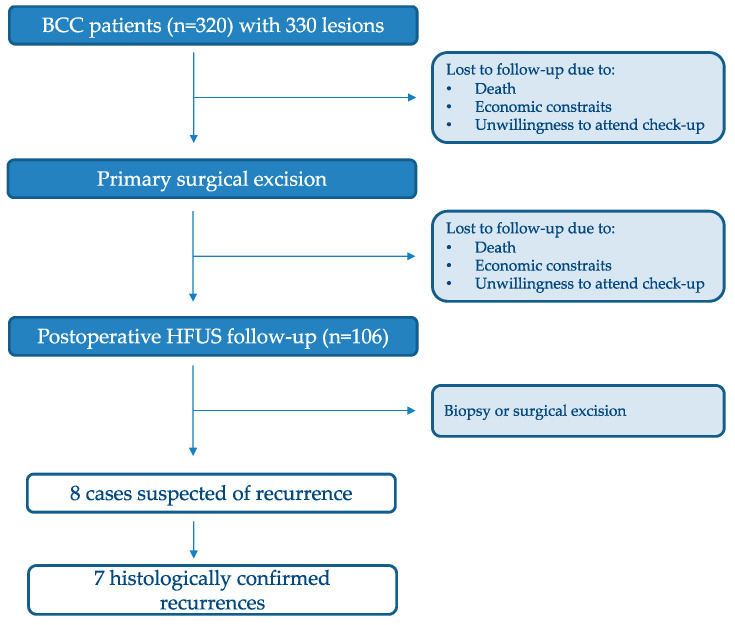
Flowchart of patient inclusion, postoperative follow-up, and recurrence detection (n = number of patients).

The study population, follow-up cohort, and recurrence analysis workflow are summarized in Figure 4.

### 2.2. Histopathological Correlation

All lesions were surgically excised and histopathologically classified. Ultrasound features were correlated with histopathological subtypes, including solid and infiltrative forms [16,19].

### 2.3. Data Analysis

Lesion morphology and vascularity were analyzed with chi-square tests. Sex- and age-stratified differences were evaluated. Odds ratios (ORs) and 95% confidence intervals (CIs) were calculated. Age groups were defined as 20–29, 30–39, 40–49, 50–59, 60–69, and ≥70 years.

## 3. Results

### 3.1. Morphology and Vascularity

**Figure 5:**
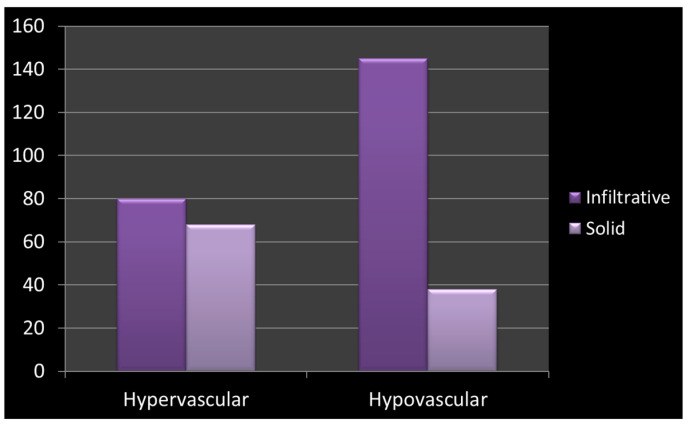
Basal cell carcinoma vascularity and morphology distribution. The *y*-axis indicates the number of cases (n). The *x*-axis shows basal cell carcinoma grouped by vascularity category (hypervascular vs. hypovascular). Bars are color coded to distinguish infiltrative morphology from solid morphology.

Morphology and vascularity demonstrated significant differences between solid and infiltrative lesions. Contour describes how clearly the boundary of a lesion can be distinguished from the surrounding tissue; solid lesions typically respect anatomic borders and therefore appear well-defined, whereas infiltrative lesions often breach or obscure these boundaries, leading to irregular or ill-defined margins. Vascularity provides additional information about perfusion and structural organization: solid lesions frequently display preserved or increased vascular supply (hypervascular), while infiltrative lesions tend to disrupt normal vascular architecture, resulting in a hypovascular appearance. Solid basal cell carcinomas were predominantly characterised by well-defined (sharp) contours, whereas infiltrative tumours typically exhibited irregular or ill-defined margins. This association was very strong, as confirmed by a high odds ratio (OR = 71.9; 95% CI: 37.0–139.8) and a highly significant chi-square test (χ^2^ = 24.7, df = 1, *p* < 0.001). Vascularity patterns differed significantly between infiltrative and solid basal cell carcinomas. Solid tumours were substantially more likely to present with hypervascular features, whereas infiltrative tumours more frequently exhibited hypovascular patterns. This association was highly significant (χ^2^ = 23.8, df = 1, *p* < 0.001), and solid lesions showed more than threefold higher odds of hypervascularity compared with infiltrative lesions (OR = 3.24). Table 1 summarizes the significant differences in contour and vascularity between solid and infiltrative lesions, demonstrating exceptionally strong associations for both parameters. Table 2 presents the distribution of hypervascular and hypovascular patterns, showing that infiltrative lesions were substantially more likely to be hypovascular compared to solid lesions. Together, these findings indicate that vascularity and contour are strongly predictive imaging features capable of distinguishing infiltrative from solid lesions with high reliability (Figure 5). These two parameters (contour and vascularity) provide high diagnostic discriminatory power and may aid in predicting lesion behaviour, aggressiveness, and potentially the need for further diagnostic or therapeutic intervention.

### 3.2. Sex-Stratified Analysis

No statistically significant difference was observed between male and female patients regarding ultrasound contour or vascularity patterns (χ^2^ test, *p* = 0.88).

### 3.3. Age-Stratified Analysis

Although descriptive trends were observed across age groups, statistical analysis revealed no significant association between age category and ultrasound contour or vascularity patterns. More than half of the elderly patients were lost to follow-up due to advanced age and comorbidities. Among those who attended regular follow-up visits, additional basal cell carcinomas developed at sites distinct from the initially detected lesion.

Table 3 summarizes the descriptive ultrasound characteristics observed across age groups. These findings should be interpreted with caution, as no statistically significant age-related differences were identified in the present cohort.

### 3.4. Recurrent Tumor Morphology and Analysis

Among the patients who participated in postoperative ultrasound follow-up, seven histologically confirmed recurrences were identified. Following primary surgical excision, 106 patients attended regular follow-up examinations with high-frequency ultrasound. Of these, eight cases were suspected of recurrence based on ultrasound findings, and seven were subsequently confirmed by histopathological examination. Recurrences were detected predominantly in the facial and cervical (neck) regions. Based on the abnormalities identified on ultrasound, biopsy or surgical excision was performed in all suspected cases. Recurrent basal cell carcinomas may be difficult to differentiate from postoperative scar tissue, which gradually becomes less dense over time. In such cases, ultrasound typically reveals an irregular hypoechoic structure at the site of previous excision. In contrast to mature scar tissue, recurrent tumors tend to maintain an irregular contour and altered echogenicity, facilitating differentiation during follow-up examinations. In the present cohort, recurrences occurred in superficial histological subtypes [20]. Due to the limited number of recurrent cases, no statistically meaningful subgroup analysis could be performed.

## 4. Discussion

High-frequency ultrasound (HFUS) provides valuable preoperative information on basal cell carcinoma morphology and vascularity, correlating closely with histopathological subtypes [9]. In the present study, distinct contour and vascular patterns differentiated infiltrative from solid lesions, which may guide surgical planning and help determine follow-up intensity [11]. Solid tumours were predominantly characterised by sharp, well-defined margins and hypervascular features, whereas infiltrative tumours more frequently exhibited irregular contours and hypovascular patterns. The strong association between ultrasound vascularity patterns and histological subtype further supports the role of high-frequency ultrasound in preoperative risk stratification and treatment planning of basal cell carcinoma. Our results align with previous research showing that HFUS features such as lesion contour and vascularity correlate with underlying histological subtype [21,22].

The present study offers novel insights compared with previously published data. To our knowledge, no earlier analysis has examined such a large patient cohort while simultaneously evaluating age, sex, ultrasound morphology, and vascularity in relation to histopathology. Although a similar study investigated vascular patterns and head-and-neck localization through dermoscopy and correlated these parameters with histopathology, ultrasound assessment was not included [23]. In contrast, despite the more limited sample size, the current well-characterized cohort demonstrates that ultrasound morphology identifies basal cell carcinomas with a greater likelihood of recurrence [24]. Recurrence analysis was based on the postoperative ultrasound follow-up cohort, enabling detection of early residual or recurrent disease.

These findings support HFUS as a complementary tool to dermatoscopy in BCC management, aiding in treatment selection and recurrence monitoring. According to the literature, postoperative ultrasound follow-up has often been performed earlier than three months; however, scar tissue typically undergoes regression after the 3-month period, meaning that recurrence can be detected with higher sensitivity once this regression has occurred [24]. Although descriptive trends were observed across sex and age groups, these differences did not reach statistical significance in the present cohort and should be interpreted with caution. In recurrent cases, ultrasound morphology and vascularity did not show a consistent correlation; rather, irregular lesion contours combined with a hypoechoic appearance were more suggestive of recurrence [20,25].

Although basal cell carcinoma very rarely produces distant metastases, recurrence may still occur even when excision is performed with adequate histological margins. For this reason, patients must be encouraged to attend regular follow-up examinations. Earlier recommendations suggested a minimum follow-up period of three years for patients with BCC [26,27]. Since then, the use of high-frequency ultrasound has significantly improved postoperative monitoring by enabling precise delineation of lesion margins and supporting accurate re-excision when necessary [28,29]. HFUS also facilitates evaluation of treatment outcomes in cases where recurrences are managed with alternative therapeutic modalities, contributing to optimized long-term care [24].

A limitation of this study was the low participation rate in follow-up examinations, which was most often attributed to economic reasons across both older and younger age groups, as well as decreased health awareness among older individuals. This loss to follow-up may have introduced selection bias and may limit the generalizability of the long-term recurrence data, as patients who returned regularly for follow-up may represent a more health-conscious or clinically stable subgroup. Notably, a low recurrence rate was observed among patients who adhered to regular follow-up, which may be partly explained by the role of the initial ultrasound examination in guiding adequate surgical excision margins. From the perspective of our clinic, ultrasound examination has become an integral component of dermatological diagnostics. Its continued development supports the differentiation of basal cell carcinomas, contributing not only to surgical planning but also to the monitoring of alternative therapeutic approaches. Furthermore, increasing patient awareness remains essential, as the clinical significance of this malignancy lies in its tendency to recur, underscoring the importance of regular follow-up. A further limitation of this study is that although examinations were initially performed in a blinded manner, the examiner inevitably accumulated additional experience correlating ultrasound morphology with histopathological findings over the course of the study period. While the examiner had substantial prior expertise before study initiation, a degree of observer learning bias cannot be completely excluded. Follow-up adherence remains a particular challenge among male patients, who may pay less attention to scars or evolving lesions and may delay seeking oncological consultation. Our findings highlight the need for thorough patient education, especially in individuals with aggressive tumor features, as this may improve compliance with follow-up recommendations in high-risk groups.

A decade of patient follow-up underscores the pivotal importance of performing a precise preoperative high-resolution ultrasound (HRUS) assessment. HRUS enables accurate delineation of lesion margins and supports the surgeon in determining an appropriate safety margin, thereby enhancing surgical precision and reducing the risk of local recurrence [14]. Nevertheless, the literature also indicates that recurrence is not inevitable even in cases where lesions were excised without histologically intact margins, underlining the multifactorial nature of recurrence risk [26,30].

Despite the growing use of high-frequency ultrasound in dermatologic oncology, several important gaps remain in the current literature. Long-term prospective studies assessing whether HFUS-guided surgical planning reduces recurrence rates are still limited. Evidence regarding the role of vascularity patterns in predicting recurrence is promising but remains inconsistent across studies, underscoring the need for standardized assessment protocols. Furthermore, although postoperative HFUS appears valuable for detecting early residual or recurrent disease, optimal follow-up intervals have not yet been uniformly defined. By addressing these gaps, future research may strengthen the integration of HFUS into evidence-based BCC management algorithms.

## 5. Conclusions

High-frequency ultrasound represents a robust and clinically valuable imaging modality for the preoperative evaluation of basal cell carcinoma. In this large, well-characterized cohort, ultrasound contour and vascularity patterns demonstrated a strong and highly significant association with histopathological subtype, enabling reliable differentiation between solid and infiltrative tumours.

Solid basal cell carcinomas were predominantly characterised by sharp, well-defined margins and hypervascular features, whereas infiltrative tumours more frequently exhibited irregular contours and hypovascular patterns. These morphological and functional characteristics may assist in surgical planning, margin selection, and risk stratification.

No statistically significant associations were observed between ultrasound morphology and patient sex or age, underscoring the importance of interpreting descriptive trends with caution. Postoperative ultrasound follow-up enabled detection of early recurrence in a subset of patients, supporting the value of HFUS in long-term surveillance.

Overall, HFUS serves as a powerful adjunct to dermatoscopy in the management of basal cell carcinoma, contributing to improved diagnostic accuracy, optimized treatment selection, and enhanced postoperative monitoring.

## Figures and Tables

**Table 1:** Comparison of Contour and Vascularity Features in Solid and Infiltrative Basal Cell Carcinomas with Corresponding Statistical Measures.

Feature	Solid Lesions	Infiltrative Lesions	Statistical Association
Contour	Mostly well-defined margins	Mostly irregular/ill-defined margins	OR = 71.9, 95% CI: 37.0–139.8 χ^2^ = 24.7, df = 1, *p* < 0.001
Vascularity	More commonly hypervascular	Strongly predisposed to hypovascularity	OR = 6.06, 95% CI: 3.51–10.46 χ^2^ = 23.8, df = 1, *p* < 0.001

**Table 2:** Frequency of Hypervascular and Hypovascular Ultrasound Patterns in Infiltrative and Solid BCC Lesions (n = the number of lesions).

Vascularity	Infiltrative (n)	Solid (n)
Hypervascular	80	67
Hypovascular	145	38

**Table 3:** Descriptive ultrasound characteristics of basal cell carcinoma by age group.

Age Group (Years)	Most Frequently Observed Ultrasound Features ^1^
20–29	Infiltrative and superficial morphology
30–39	Solid, nodular, and adenomatous morphology
40–49	Increased frequency of hypervascular lesions
50–59	Mixed vascularity patterns
60–69	Increased frequency of irregular contours
≥70	Combined presence of irregular contour and hypervascularity

^1^ No statistically significant association was observed between age group and ultrasound contour or vascularity patterns.

## Data Availability

Data supporting the findings of this study are restricted due to GDPR regulations. They can be made available by the authors upon justified request and in accordance with institutional and ethical guidelines.
